# Evaluation of renal cortical echogenicity in healthy cats using anisotropic backscatter artifacts and echogenicity differences among internal organs

**DOI:** 10.1080/01652176.2023.2177773

**Published:** 2023-02-13

**Authors:** Gong-Yi Yan, Pin-Chen Liu, Ming-Jen Kang, Wen-Sheng Li, Yen-Huan Lee, Kuan-Sheng Chen

**Affiliations:** a Department of Veterinary Medicine, College of Veterinary Medicine, National Chung Hsing University, Taichung, Taiwan; b Veterinary Medical Teaching Hospital, College of Veterinary Medicine, National Chung Hsing University, Taichung, Taiwan

**Keywords:** Cat, feline, echogenicity, anisotropy, insonation, backscatter artifact, renal cortex, liver, spleen

## Abstract

**Background:**

Renal cortical anisotropic backscatter artifact (CABA) is a hyperechoic region of the renal poles where the insonation of sound beams is perpendicular to the renal tubules within the renal cortex.

**Aims:**

To determine whether renal CABA can be observed in healthy cats and to compare the echogenicity of renal CABA with that of the spleen and liver.

**Material and methods:**

Images of the spleen, liver, kidneys, and urinary bladder were acquired from 30 clinically healthy cats with renal CABA. Echogenicity differences among organs and echo scores within urine were recorded and analyzed. All ultrasound images were acquired using a 7.2–14-MHz linear transducer. Univariate logistic regression was used to assess the associations between the presence of renal CABA and various variables.

**Results:**

The prevalence of the renal CABA was 86.7% (26/30) and 93.3% (28/30) according to different observers. The reproducibility of renal CABA is substantial to excellent. The renal CABA echogenicity was greater or equal to the spleen and greater than the hepatic echogenicity in 90.0% of cats (27/30). For comparison with the spleen and liver, there were three and six combinations of echogenicity differences using the CABA and non-CABA regions, respectively. The renal cortical echogenicity in the CABA region was higher than the liver in all subjects. Renal CABA was not associated with age, body weight, gender, body condition score, or lipid droplets in the urinary bladder.

**Conclusion:**

Renal CABA was present in most healthy cats and could be used for echogenicity comparisons with the liver and spleen.

## 1. Introduction

Ultrasonography is an excellent non-invasive diagnostic modality for visualizing renal architecture, as well as providing quantitative and subjective evaluations of the feline kidneys (Debruyn et al. 2012). The renal size, contour, corticomedullary junction or echogenicity, which can assist in the detection of kidney diseases, may be altered in renal inflammation, tumors, or systemic diseases (Griffin 2020). Renal cortical echogenicity may be hyperechoic due to fat deposition within the cortical tubular epithelium (Yeager and Anderson 1989).

Renal cortical echogenicity is often compared with spleen and liver echogenicity in veterinary medicine (Drost et al. 2000; Yabuki et al. 2008; Sayre and Spaulding 2014). However, it is assumed that the organs used for comparison are normal (Banzato et al. 2016). Different cortical regions used for organ echogenicity comparisons could result in differences in echogenicity between the organs (Ivančić and Mai 2008).

The echogenicity of organs varies based on the orientation of the tissue relative to the insonation angle of the ultrasound beam; this phenomenon is an anisotropic backscatter artifact (Rubin et al. 1988). It can be observed in different organs and has been described in many species (e.g. sheep, dogs, cats, and swine) (Rubin et al. 1988; Insana et al. 1991; Sayre and Spaulding 2014).

The renal cortical anisotropic backscatter artifact (CABA) is formed by directionally dependent insonation angles of sound waves relative to the renal tubules and is observed in the renal cortex (Insana et al. 1991). It is mainly generated from the proximal straight tubules, loops of Henle, and collecting ducts oriented perpendicular to the renal capsules (Rubin et al. 1988; Insana et al. 1991). Anisotropic artifacts predominantly appear in the renal cortex because the renal tubules in the medulla are thinner and more tightly packed than those in the cortex (Rubin et al. 1988). Renal CABA is located in the 3 and 9 o’clock positions within the renal cortex (renal pole), and best appreciated in median longitudinal scanner as a hyperechoic area with ill-defined margins (Rubin et al. 1988). The absence of renal CABA has been found to be significantly associated with feline chronic kidney disease stages and could be used as an alternative method for its evaluation (Chou et al. 2021).

We hypothesized that renal CABA would be present in the renal cortex in clinically healthy cats, and that the presence of renal CABA was not associated with body weight, body condition score (BCS), age, gender, reproductive status, or the presence of echoes with urine or lipid droplets within urine. Hence, the objectives of this study were to determine if renal CABA was present in clinically healthy cats and to evaluate the echogenicity differences between the liver, spleen, and renal cortex from CABA regions.

## 2. Materials and methods

In this prospective study, adult clinically healthy cats owned by students and staff at the Veterinary Medical Teaching Hospital, NCHU, were recruited with the owner’s consent from 2020 to 2022.

The inclusion criteria were as follows: (1) clinically healthy cats; (2) no known history of hepatic, renal, or lower urinary tract disease; and (3) no medication used except for ectoparasite or endoparasite prevention drugs in the past 6 months. Characteristics such as gender, age, body weight, and breed were recorded for all cats, and they underwent physical examination, hematocrit measurement, serum biochemical assessments (alanine transaminase [ALT], aspartate aminotransferase [AST], alkaline phosphatase [ALKP], total bilirubin, creatinine, blood urea nitrogen, symmetric dimethylarginine [SDMA], albumin), ultrasound examinations, and ultrasound-guided cystocentesis for routine urinalysis. In addition, the body condition score (BCS) of the cats was evaluated according to the 9-point BCS system (Bjornvad et al. 2011).

Cats with the following results were excluded: anemia (hematocrit <30%), serum ALT activity >187 U/L, serum AST activity >60 U/L, serum ALKP activity >92 U/L, serum total bilirubin concentration >8.6 µmol/L, serum albumin <23 g/L, serum SDMA concentration >0.69 µmol/L, proteinuria (urine protein/creatinine ratio >0.2), hematuria (>5 erythrocytes/high-power field), suspected urinary tract infection (>5 white blood cells/high power field or bacteria seen in the urinary sediment), or splenic, hepatic or renal abnormalities (e.g. focal or multifocal lesions, enlargement or irregular margins of these organs, hydronephrosis, renal hypoplasia, perirenal or peritoneal effusion) on ultrasound examination.

The cats were fasted for approximately 8 h before the ultrasound examination. Cats were manually restrained in lateral or dorsal recumbency throughout the ultrasound procedure.

All ultrasound images and cine loop clips of the spleen, liver, kidneys, and urinary bladder were acquired using an ultrasound machine (Xario SSA-660A; Toshiba Medical Systems Corporation, Minato, Tokyo, Japan) with a 7.2–14-MHz linear transducer (PLT-1204 BT; Toshiba Medical Systems Corporation, Minato, Tokyo, Japan), and retrieved from the Picture Archiving and Communication System in the teaching hospital. Abdominal ultrasonography was performed by an experienced radiologist (KSC) with 18 years of experience in ultrasonography.

The kidneys were imaged in longitudinal and transverse planes. For organ echogenicity comparison in each subject, the twin-view display was used if two organs could not be placed in one view. The comparison of the echogenicity between renal cortex and the spleen and hepatic parenchyma was performed twice with the focal zone first placed at the renal CABA and then at the non-CABA region. The settings for focal zone, gain and image depth were the same in the twin-view display for organ comparison.

At least 3 ml of urine was collected using ultrasound-guided cystocentesis. The sample was subjected to routine urinalysis (color, turbidity, specific gravity, dipstick analysis, urine protein/creatinine ratio and sediments) within 20 min after collection.

**Figure 1.**
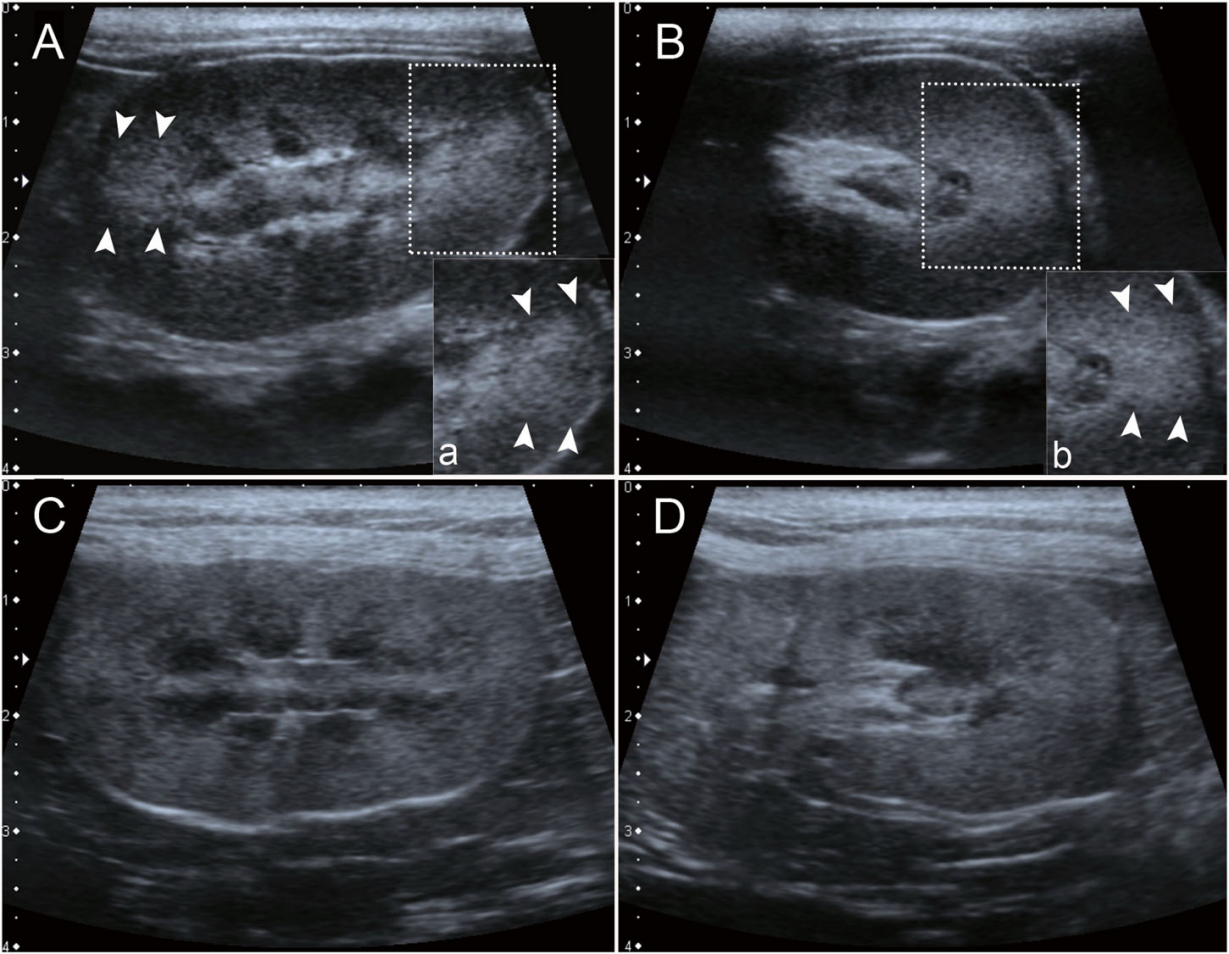
The presence (A, B) and absence (C, D) of renal CABA in median longitudinal (A, C) and transverse (B, D) plane of the feline kidney. Magnified images of the presence of renal CABA from the rectangular dotted region of (A) and (B) are shown in (a) and (b), respectively. The arrowheads indicate focal hyperechogenic trapezoid regions with indistinct or faded margins at 3 and 9 o’clock of the renal cortex compared with the rest of the cortex.

A third-year radiology resident (observer 1) and a first-year radiology postgraduate student (observer 2) were assigned to evaluate the presence or absence of renal CABA (Figure 1), as previously described (Chou et al. 2021). Observer 1 and 2 were trained for 2.5 years and allowed 20 min to familiarize themselves with the presence and absence of renal CABA, respectively. Both observers used the same medical image viewer software (v.3.3.6; The Horos Project) on the same monitor. To avoid interpretation bias, both observers were blinded to the objective and hypothesis of the study and to the information of the subjects. Furthermore, images of absent renal CABA from 20 cats with chronic renal disease were mixed with renal images from the recruited subjects for evaluation. The subject was considered to have renal CABA when CABA was observed in one or both kidneys. Each observer performed the evaluation within a 7-day interval.

All procedures were approved by the Veterinary Medical Teaching Hospital and Institutional Animal Care and Use Committee of the National Chung Hsing University (NCHU; no. 108-104 and 109-151).

**Figure 2.**
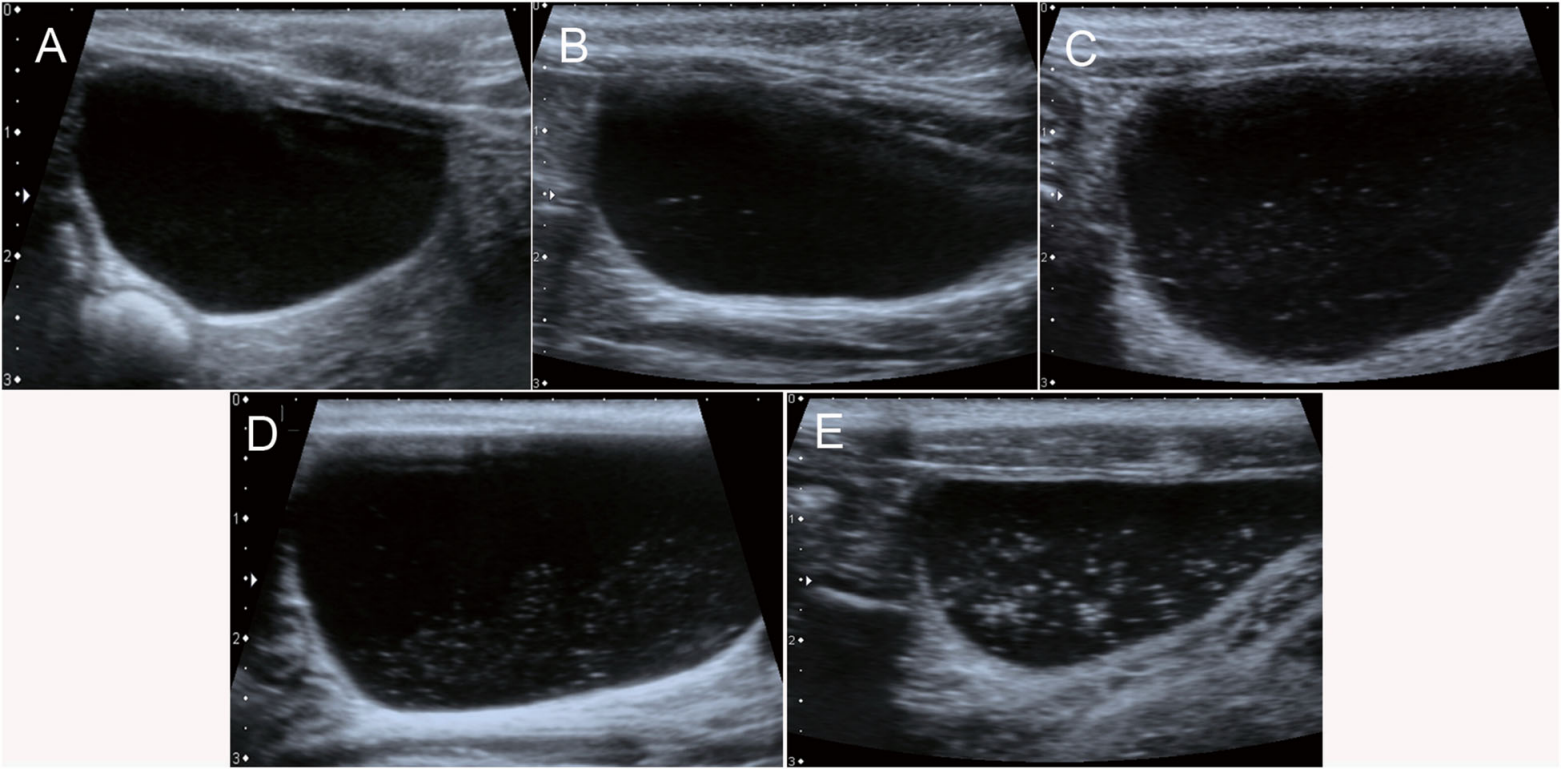
Illustration of echo scores within urinary bladder. The urinary bladder with anechoic urine was scored as 0 (A); less than 10 echoes was scored as 1(B); echoes that occupied less than 25% of the lumen was scored as 2 (C); echoes that occupied 25–50% was scored as 3 (D); echoes that occupied more than 50% was scored as 4 (E).

As previously described (Sislak et al. 2014), the number of echoes identified within the urine was subjectively scored as 0, 1, 2, 3, and 4 to represent none, few, mild, moderate, or many echoes, respectively (Figure 2). No echo observed in anechoic urine was considered none. Urinary bladders with less than 10 discrete echoes were considered few. Echoes that occupied less than 25%, 25–50%, and more than 50% of the lumen was considered mild, moderate, and many, respectively.

**Figure 3.**
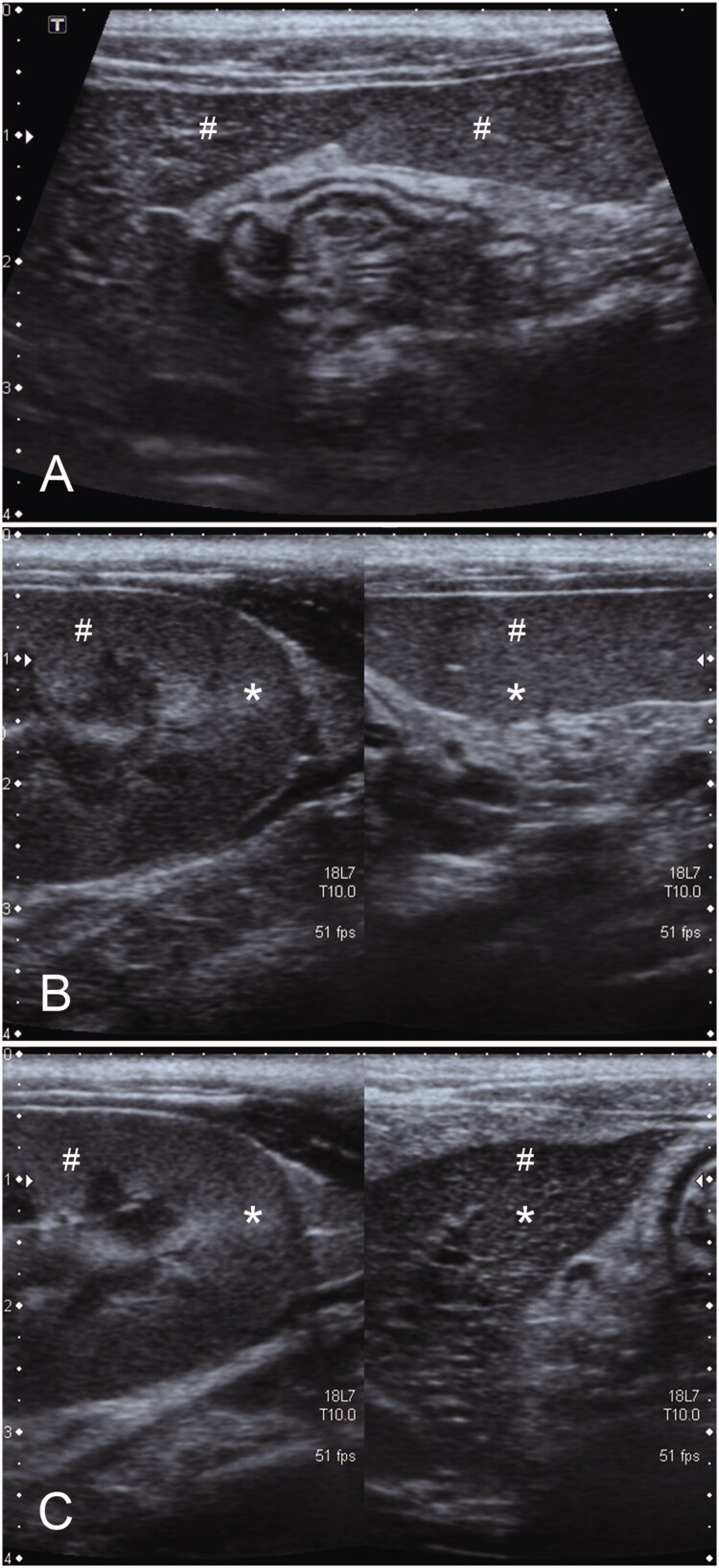
Illustrations of the tissue comparisons between CABA (*) and non-CABA region (#). Hepatic versus splenic echogenicity (A), renal cortex versus splenic echogenicity (B), and renal cortex versus hepatic echogenicity (C). CABA, cortical anisotropic backscatter artifact.

Evaluation of the differences in echogenicity among the spleen, liver, and renal cortex, from the most to the least echogenic, was assessed by consensus. Renal echogenicity was evaluated in renal and non-CABA regions (Figure 3).

All statistical analyses were performed using SAS software (version 9.4; SAS Institute Inc., Cary, NC, USA). Data were assessed for normality using the Shapiro–Wilk test. The mean and standard deviation values were calculated for normally distributed data, whereas non-normally distributed data were reported as median (range).

Fisher’s exact test was used to determine whether there was a significant association between the presence of renal CABA and different categorical variables, including gender, reproductive state, presence of lipid droplets in urine, or presence of echoes within the urinary bladder, and between the presence of urine lipid droplets and the presence of urinary bladder echoes. Univariate logistic regression was used to assess the associations between the presence of renal CABA and variables including age, body weight, BCS, serum creatinine or serum SDMA. Intra- and inter-observer agreement of the presence of renal CABA were assessed using McNemar’s test and Cohen’s Kappa, and the weighted Kappa was used to assess agreement between observers in the evaluation of echoes in the urinary bladder. The definition of Cohen’s Kappa value is >0.80 (excellent), 0.61-0.8 (substantial), and 0.41–0.6 (moderate) (Landis and Koch 1977). Statistical significance was set at *p* < 0.05.

## 3. Results

Thirty clinically healthy cats were included in this study. The clinical data are summarized in Table 1. All cats tested negative for feline leukemia and feline immunodeficiency viruses. No complications were observed after ultrasound-guided cystocentesis in any cat.

**Table 1.** Clinical data of all included cats.

Clinical data	
Age (years)	4.5 (1–10; IQR, 2.0–6.0)
Gender	
Intact female	14
Intact male	5
Female spayed	3
Male neutered	8
Breed	10 mixed cats, 5 Persians, 4 Ragdolls, 4 Munchkins, 3 American shorthairs, 2 British shorthairs, 1 Exotic shorthair, 1 Norwegian forest
Body weight (kg)	3.8 ± 1.4
Serum creatinine (µmol/L)	97.2 (53.0–141.4; IQR, 88.4–132.6)
Serum SDMA (µmol/L)	0.55 (0.40–0.69; IQR 0.45–0.64)
Body condition score	4.0 (3–9; IQR 4–5)

Note: Age, serum creatinine, serum SDMA and body condition score were present as median (range). Body weight was presented as mean ± SD. IQR: Interquartile range.

Each observer assessed the presence of renal CABA twice. Intra-observer evaluation of the presence of renal CABA for each observer showed agreement (*p* = 0.32 for each observer’s evaluation, McNemar test) and had a Cohen’s Kappa value (agreement) of 0.87 (excellent) and 0.78 (substantial) for observer 1 and 2, respectively. Inter-observer agreement for the presence of renal CABA in the first and second evaluations was also in agreement (*p* = 0.16 for each evaluation, McNemar test) with Cohen’s Kappa 0.63 (substantial) and 0.71 (substantial), respectively. The prevalence of renal CABA in our study was 86.7% (26/30) and 93.3% (28/30), according to the first results from observers 1 and 2, respectively.

For scoring the echoes within the bladder, the weighted Kappa value was 0.88 (excellent) for each observer. Inter-observer agreement for scoring the echoes within the bladder in the first and second time was 0.84 (excellent) and 0.85 (excellent), respectively. Echoes within the urinary bladder were observed in 83.3% (25/30) of the clinically healthy cats, lipid droplets were identified in urine sediments of 17/30 cats (56.7%). No abnormalities were found in specific gravity or urine protein: creatinine ratio in any of the cats.

In Fisher’s exact test, there was no statistically significant difference between the presence of renal CABA and different categorical variables. In the univariate analysis, none of the variables were associated with the presence of renal CABA (Table 2). The association between the presence of urinary bladder echoes and lipid droplets in the urine was not statistically significant (*p* = 0.13)

**Table 2.** Associations among the variables with the presence of renal CABA from different observers.

	The presence of renal CABA	
	Observer 1	Observer 2
Gender	*p* = 1.00	*p* = 0.49
Reproductive state	*p* = 1.00	*p* = 0.54
Presence of lipid droplets in urine	*p* = 0.29	*p* = 1.00
Presence of echoes within bladder	*p* = 1.00	*p* = 1.00
Age	*p* = 0.72	*p* = 0.35
Body weight	*p* = 0.87	*p* = 0.41
BCS	*p* = 0.78	*p* = 0.70
Serum SDMA	*p* = 0.71	*p* = 0.93
Serum creatinine	*p* = 0.88	*p* = 0.56

Gender, reproductive state, presence of lipid droplets in urine and presence of echoes within bladder were analyzed by Fisher’s exact test. Age, body weight, BCS, serum SDMA and serum creatinine were analyzed by univariable analysis. Statistical significance *p* < 0.05. CABA: Cortical anisotropic backscatter artifact; BCS: Body condition score; SDMA: Symmetric dimethylarginine.

A comparison of echogenicity differences among the kidney, spleen, and liver using CABA and non-CABA regions was performed (Table 3). No echogenicity difference existed in either kidney in any of the subjects. There were three and six combinations of echogenicity differences using the CABA and non-CABA region, respectively, for comparison with the spleen and liver. The renal echogenicity was either greater (56.7%) or similar to the spleen (33.3%), using the CABA region for comparison. The most common combination (43.3%) was the renal echogenicity similar to the spleen but greater than the liver, using the non-CABA region for comparison. The renal cortical echogenicity in the CABA region was higher than the liver in all subjects (100%).

**Table 3.** Comparison of echogenicity differences among spleen, liver and renal cortex at CABA region and non-CABA region.

Echogenicity	CABA region	non-CABA region
Kidney > Spleen > Liver	56.7% (17/30)	16.7% (5/30)
Kidney = Spleen > Liver	33.3% (10/30)	43.3% (13/30)
Spleen > Kidney > Liver	10.0% (3/30)	20.0% (6/30)
Spleen > Liver > Kidney		13.3% (4/30)
Spleen = Liver > Kidney		3.3% (1/30)
Liver > Kidney > Spleen		3.3% (1/30)

CABA: Cortical anisotropic backscatter artifact.

## 4. Discussion

This prospective study illustrated that the presence of renal CABA on ultrasonography is common in clinically healthy cats. The renal CABA was observed in 86.7% and 93.3% of the clinically normal subjects by two observers in our study. These results support our hypothesis that renal CABA would be present in the renal cortex of feline kidneys with normal renal function based on serum SDMA and creatinine concentrations. Similar to a previous study (Chou et al. 2021), the presence of renal CABA was observed in 88.9% of clinically healthy cats with serum creatinine <141.4 µmol/L. The presence of renal CABA, which could be observed in most healthy cats, could be used as a reference to assess the normal renal cortex.

Intra-observer agreement in this study from two observers was substantial and excellent, similar to the agreement described previously (Chou et al. 2021). This suggests that the level of training and experience in ultrasonography did not make a significant difference in evaluating the presence of renal CABA, making it a repeatable and reproducible tool for evaluating renal cortical echogenicity, as previously described (Chou et al. 2021).

Different regions of the renal cortex were chosen for internal organ comparison in this study (Table 3). Using the CABA region for comparison appeared to be better because it had fewer organ echogenicity combinations than the non-CABA region in this study and a previous study (five combinations) (Sayre and Spaulding 2014). Moreover, 90.0% (27/30) and 60.0% (18/30) of the population in this study showed that the renal cortex was hyper- or isoechogenic to the spleen and hyperechogenic to the liver using CABA region and non-CABA region, respectively, indicating that echogenicity of the kidney ≥ spleen > liver in most of the healthy cats using CABA region for comparison.

Previous study using the renal cortical region (non-CABA region) showed that the proportion of clinically healthy cats with the renal cortical echogenicity greater or equal to spleen was 93.5% (Sayre and Spaulding 2014), which was greater than that in this study (60.0%). The difference between these two results may be due to the differences in the ultrasound machines used in the studies.

When using CABA region for comparison, the renal echogenicity was greater than the liver (100%), but the percentage decreased to 80.0% when using the non-CABA region for comparison. Similar to a study in dogs, the echogenicity of the right cranial pole of the kidney has been described to be significantly greater than the adjacent liver in 88%. However, the percentage decreased to 32% in the near field region within the kidney using harmonic mode (Ivančić and Mai 2008). The echogenicity difference in dogs suggests an effect of anisotropy. Therefore, the region selected within the kidney for organ echogenicity should be carefully selected.

This study provided the echogenicity differences among internal organs by evaluating the renal CABA and non-CABA regions. However, a comparison of echogenicity differences among internal organs may have some disadvantages. The relative echogenicity comparison between organs assumes that the organs for comparison are clinically normal (Banzato et al. 2016). In contrast, the evaluation of the presence of renal CABA is an auto-referencing method without the disadvantages described above.

In this study, echoes within the urinary bladder were seen in 83.3% of the clinically healthy cats, while lipid droplets in urine were identified in 56.7%. There was no association between echoes in the urinary bladder and the presence of lipid droplets in the urine. These results were similar to those in the previous study (Sislak et al. 2014) where echoes in the urinary bladder was observed in 90% of the healthy cats, 66% of which showed unremarkable urinalysis results except for the presence of lipid droplets in the urine. Likewise, Sislak et al. (2014) also reported no association between the presence of urinary bladder echoes and lipid droplets in the urine. One potential explanation for these findings might be that the constituents (diacylglycerol, free fatty acids, triglycerides, and cholesterol ester) of urine lipids vary and only diacylglycerol, but not the rest of the lipid subfractions, had been shown to associate with echoes in the bladder (Sislak et al. 2014).

Previous studies have shown that lipid deposition in the proximal tubular epithelium increased in the renal cortex in cats (Yeager and Anderson 1989; Sislak et al. 2014), and that lipiduria is correlated with shedding of lipid from the tubular epithelium (Sislak et al. 2014; Schwarz et al. 2021). It appears that the echogenicity of non-CABA region was used for the comparison of echogenicity differences among internal organs in the previous study (Sayre and Spaulding 2014), and this could be influenced by lipid deposition in the renal cortex. In this study, the renal CABA was not associated with the urine lipids or echoes in the urinary bladder, suggesting that the lipid deposition in the tubular epithelium may not affect the incidence of ultrasound beam reflection from the renal tubules that are oriented perpendicular to the ultrasound beam. Thus, evaluating the presence of renal CABA or using the CABA region for organ echogenicity comparison may not be influenced by the lipid deposition in the cortex.

The major limitations of this study were the relatively small sample size and that histopathological examination of the spleen, liver, and kidney was not performed. These organs were defined as clinically healthy based on their history, physical examination findings, and clinicopathology. Although histopathological results were not obtained in this study, the results from clinicopathological examinations could exclude most diseases in these organs.

In conclusion, this study provides two alternative methods for evaluating the normal feline cortex. One is using the presence of renal CABA as auto-reference, which is present in >90% of healthy cats. The other was using renal CABA region for internal organ comparison, with echogenicity kidney ≥ spleen > liver found in 90.0% of clinically healthy cats. Evaluation of the presence of renal CABA showed substantial to excellent repeatability and reproducibility, making this subjective method reliable. It appears that the presence of renal CABA was not influenced by age, body weight, gender, BCS, echoes in the urinary bladder, or the amount of fat droplets deposited within the urinary bladder. Nevertheless, further studies should be performed to validate these findings. These two methods should be used in conjunction with clinicopathological examinations.
